# Association of *CTXN3-SLC12A2* polymorphisms and schizophrenia in a Thai population

**DOI:** 10.1186/1744-9081-8-27

**Published:** 2012-05-29

**Authors:** Benjaporn Panichareon, Kazuhiro Nakayama, Sadahiko Iwamoto, Wanpen Thurakitwannakarn, Wasana Sukhumsirichart

**Affiliations:** 1 Department of Biochemistry, Faculty of Medicine, Srinakharinwirot University, Bangkok, 10110, Thailand; 2 Division of Human Genetics, Center for Molecular Medicine, Jichi Medical University, Tochigi, 329-0498, Japan; 3 Department of Psychiatry, Faculty of Medicine, Srinakharinwirot University, Bangkok, 10110, Thailand

**Keywords:** Schizophrenia, Single nucleotide polymorphisms, *CTXN3*, *SLC12A2*, High- resolution melting analysis

## Abstract

**Background:**

A genome-wide association study (GWAS) combined with brain imaging as a quantitative trait analysis revealed that the SNPs near *CTXN3-SLC12A2* region were related to forebrain development and stress response which involved in schizophrenia. In the present study, the SNPs in this region were analyzed for association with schizophrenia in a Thai population.

**Methods:**

A total of 115 schizophrenia and 173 unrelated normal controls with mean age of 37.87 ± 11.8 and 42.81 ± 6.0 years, respectively, were included in this study. Genotyping was performed using polymerase chain reaction and high-resolution melting (HRM) analysis. The difference in genotype distribution between patient and control was assessed by Chi-square test of the SPSS software.

**Results:**

We found a significant association between the GWAS-discovered SNP, rs245178, with the risk of schizophrenia in the Thai population [*P* = 0.006, odds ratio for the minor G allele: 0.62(0.46–0.83)]. Additionally, another potential SNP, rs698172, which was in moderate linkage disequilibrium with rs245178, also showed strong association with schizophrenia [*P* = 0.003, odds ratio for minor T allele: 0.61(0.46–0.82)]. This association remained significant at 5% level after the Bonferroni correction for multiple testing.

**Conclusions:**

This study shows that two SNPs in intergenic of the *CTXN3* and *SLC12A2* genes, rs245178 and rs698172, are associated with risk of schizophrenia in Thai population. Further study is required for clarification the role of genetic variation around these SNPs in expression pattern of the *CTXN3* and *SLC12A2* genes, which may be involved in schizophrenia pathogenesis.

## Background

Schizophrenia is characterized by abnormalities in perception, thought, cognition, and expression of reality. It is clinically described by positive and negative symptoms [1]. Positive symptoms are psychotic behaviors that are present in schizophrenia but absent in healthy individuals, include hallucinations, delusions, and thought disorder. Negative symptoms are factors that absent in schizophrenia patients but are present in healthy persons [2]. Schizophrenia is one of the most common psychiatric disorders and its prevalence is 1% of the worldwide population [3]. Twin and family studies suggest that genetic factors play a major role in schizophrenia [4,5].

A variety of genetic studies reported several chromosomal regions associated with schizophrenia and related phenotypes. A quantitative trait locus for schizophrenia was mapped to a chromosome 5 region in Americans of European ancestry [6]. A meta-analysis of European ethnicity showed that the 5q23.2-q34 region was significantly linked with schizophrenia [7].

A genome wide association study (GWAS) in combination with the data from brain imaging of dorsolateral prefrontal cortex (DLPFC) as quantitative trait (QT) analysis revealed 6 genes/regions that involved in neurodevelopment and response to stress. The first three genes including *ROBO1-ROBO2TNIK*, and *CTXN3-SLC12A2* were identified by having 2 single-nucleotide polymorphisms (SNPs) each significant at *P* < 10^−6^ for the interaction between the imaging QT and the diagnosis. The other three genes including *POU3F2TRAF*, and *GPC1* had a significant SNP at <10^−6^[8]. Because the GWAS was a screening method over the whole genome to identify the SNPs related to risk for schizophrenia and the subject of previous study mostly Caucasian ethnic group [8]. Moreover, there is no association study of those genes and risk of schizophrenia in other ethnic groups rather than European ancestry. A replication study with Asian ethnic groups would help to reinforce the signals obtained in a European GWAS. Furthermore, the testing of a population showing different linkage disequilibrium (LD) structures may assist in identifying the true causative variant in the genomic region of interest.

In Thailand, the prevalence of schizophrenia is comparable with the worldwide value (approximately 1%). Genetic variations associated with risks of schizophrenia have been investigated in opioid-binding protein/cell adhesion molecule (OPCML) gene [9] using GWAS-discovered SNPs of Europeans ancestry [10]. There was strong association between an intronic SNP of the *OPCML* gene (rs1784519) and the risk of schizophrenia in a Thai population *P* = 0.00036, odds ratio for the minor A allele: 2.11(1.57–2.84)] [9].

This study aims to investigate the association between SNPs in *CTXN3-SLC12A2* region with the risk of schizophrenia in the Thai population. The *CTXN3* gene is located on 5q23 encode CTXN3 (cortexin 3), a brain specific integral membrane protein highly enriched in cortex. It is expressed in fetal brain and increases in density perinatally. The gene consists of 3 exons and spans an approximate 9.6 kb region. It has 2 alternative transcript variants expressed in the brain and kidneys, and encodes an 81-amino acids protein [11]. This gene is highly conserved in various species, suggesting that this gene plays important roles in brain and kidney function. The gene, *SLC12A2* (solute carrier family 12, member 2), found in the same region as CTXN3, encodes a sodium-potassium-chloride (Na-K-Cl) cotransporter protein which is expressed in various human tissues, including the brain [12]. SLC12A2 is involved in the regulation of GABA neurotransmission. Both CTXN3 and SLC12A2 are indicated to play a role in normal brain function, in addition, the *CTXN3-SLC12A2* region is considered as the second most important region linked to schizophrenia in the meta-analysis [7,8], therefore, they may be involved in schizophrenia pathogenesis. This study, the SNPs in the intergenic of *CTXN3-SLC12A2* region, 3′-UTR and intronic of *CTXN3* gene were analyze for their association with schizophrenia.

## Methods

### Subjects

A total number of subjects consisted of 115 schizophrenia patients (61 males and 54 females) with a mean age of 37.87 ± 11.8 years and 173 unrelated healthy controls (110 males and 63 females) with a mean age of 42.81 ± 6.0 years. The patients were diagnosed according to the Diagnostic and Statistical Manual of Mental Disorders, Fourth Edition (DSM-IV) criteria. The healthy controls were also psychiatrically screened to confirm that they did not suffer from a psychiatric disorder. All patients and healthy controls gave informed consent for participation in this study, which was approved by the ethical committee at the Faculty of Medicine, Chiang Mai University and Faculty of Medicine, Srinakharinwirot University.

### Genotyping

Genomic DNAs of patient and control were extracted from whole blood (5 ml) using Flexigene DNA kit (Qiagen, German). Genotyping was performed by using polymerase chain reaction (PCR) and followed by high-resolution melting analysis (HRM). PCR primers were designed using LightScanner Primer Design software (Idaho Technology, Salt Lake City, UT, USA) and the primer sequences were included in supplement table (available online). The PCR was carried out using TaKaRa *Ex Taq* Hot Start Version (TaKaRa, Tokyo, Japan) with LCGreen + (Idaho Technology). The high-resolution melting analysis was performed using a LightScanner (Idaho Technology, USA). Sequences of primers are provided in the Additional file 1.

### Statistical analysis

The linkage disequilibrium (LD) status of the GWAS-discovered marker SNPs; rs245178, rs245201 [8], and nearby SNPs; rs1421746, rs711360, rs698171, rs698172, rs245314, rs245311, rs245310, rs9285907, rs151849, rs6874357, rs245199, rs245195, rs245192, rs245191, rs1579284, rs181746, rs245186, rs6864687, rs151879, rs245179, rs245181, and rs245183, were determined using 47 randomly selected schizophrenia patients and healthy controls. LD analyses were performed using Haploview software [13]. SNPs with a marked deviation from Hardy-Weinberg equilibrium (*P* < 0.01) were excluded from further analysis. The association analysis was assessed by using Pearson’s Chi-square test implemented in SPSS program version 11.5 for Window. We assessed 3 genotype models (additive, dominant, and recessive) in the association analysis. The significance level of the association test was set at 5% and moreover, we also applied the Bonferroni correction for the numbers of SNPs tested. For the SNPs showing significant association, mode of inheritance was inferred by using the method proposed Zintzaras and Santos [14]. The generalized odds ratio of genotypes [15] and odds ratio of the minor allele of each SNP were calculated. For the allelic test, we evaluated statistical power by using the G*Power 3 [16]. Odds ratios that ensured 80% power (*P* < 0.05) were estimated as 2.00(0.37), 1.74(0.51), 1.65(0.57), 1.62(0.60), and 1.62(0.61), for alleles with frequencies in the controls of 0.1, 0.2, 0.3, 0.4 and 0.5, respectively. We also estimated sex and age adjusted-odds ratio of the SNP genotypes with the aid of multiple logistic regression analysis. Haplotypes of the SNPs were reconstructed by using PHASE2.0 [17]. Differences in haplotype frequencies between the patients and the controls were assessed by using *χ*^2^ test.

## Results

### Association study of SNPs and schizophrenia

We first tested the associations between the GWAS-discovered SNPs that located in the intergenic of *CTXN3SLC12A2* region, 3'-UTR and intronic of *CTXN3* gene with schizophrenia in Thai population. The result of the association analysis is summarized in Table 1. The SNPs in intergenic, rs245178, showed significant differences in the genotype distribution between patients and controls (*P* = 0.006, under the dominant model). The odds ratio (OR) and 95% confidence intervals (CI) of the minor G allele were estimated to be 0.62 and 0.46–0.83, respectively. The lower prevalence of the G allele in patients was consistent with the result of the previous report [8]. In contrast, another GWAS-discovered SNP, rs245201, did not show significant differences in genotype distribution in Thai population (*P* > 0.05).

**Table 1:** Results of the association analyses

**dbSNP ID**	**Type of SNP**		**Genotype frequencies**			**MAF**^**1**^	***P***^**2**^		**Allelic OR**^**3**^** Generalized OR**
rs7711139	*CTXN3*		A/A	A/G	G/G		a	0.139	1.29(0.96–1.74)
	intronic	Patients	17	45	52	0.34	d	0.528	1.23(0.84–1.95)
		Controls	13	74	85	0.29	r	0.047	
rs248707	*CTXN3*		C/C	C/T	T/T		a	0.594	1.24(0.88–1.74)
	3′UTR	Patients	7	40	67	0.24	d	0.325	1.27(0.80–2.00)
		Controls	8	53	111	0.20	r	0.580	
rs1421746	Intergenic		C/C	C/T	T/T		a	0.025	0.61(0.46–0.81)
		Patients	37	49	26	0.45	d	0.038	0.58(0.39–0.86)
		Controls	38	71	64	0.57	r	0.015	
rs698172	Intergenic		C/C	C/T	T/T		a	0.012	0.61(0.46–0.82)
		Patients	49	41	21	0.38	d	0.003*	0.58(0.38–0.87)
		Controls	47	81	45	0.49	r	0.167	
rs698171	Intergenic		G/G	G/T	T/T		a	0.028	1.56(1.13–2.16)
		Patients	57	44	11	0.29	d	0.10	1.58(1.01–2.48)
		Controls	105	63	5	0.21	r	0.01	
rs245201	Intergenic		A/A	A/G	G/G		a	0.083	0.81(0.60–1.08)
		Patients	51	41	22	0.37	d	0.052	0.77(0.51–1.15)
		Controls	56	82	30	0.42	r	0.759	
rs245178	Intergenic		A/A	A/G	G/G		a	0.021	0.62(0.46–0.83)
		Patients	54	43	15	0.33	d	0.006^*^	0.57(0.38–0.86)
		Controls	55	83	34	0.44	r	0.165	

^1^MAF: Minor allele frequency.

^2^*P* values for the additive model (a), dominant model (d), and recessive model (r) are shown. Asterisks indicate tests that were significant at 5% level after the Bonferroni correction for multiple SNP testing.

^3^Allelic OR (upper) indicates odds ratio and 95% confidence interval of minor alleles. Generalized OR (lower) indicates odds ratio and 95% confidence interval of genotypes [14].

### Linkage disequilibrium analysis

**Figure 1:**
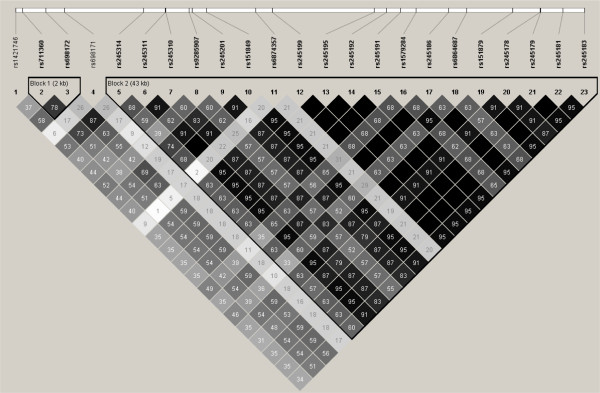
**Linkage disequilibrium status of the *CTXN3*-*SLC12A2* region in Thai individuals. ***r*^*2*^ values calculated from genotype data of 47 Thai are indicated. The haplotype blocks were defined by using the default setting of the Haploview software.

**Figure 2:**
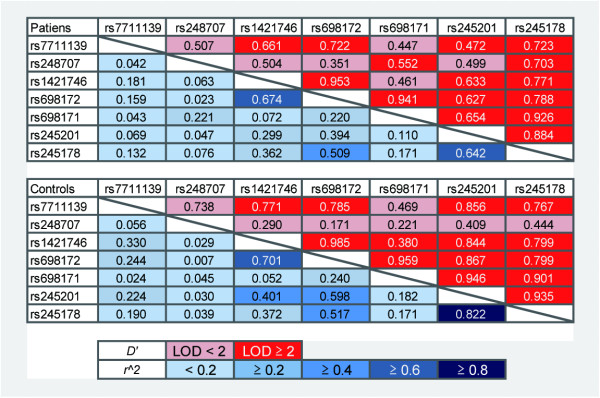
**Pairwise LD status of the 7 SNPs in 115 patients and 173 controls.** Upper and lower triangles indicate *D’* and *r*^*2*^, respectively. LD status of each pair of SNPs is color-coded by its strength (see the explanatory notes in the figure).

The LD status of rs245178 and nearby SNPs in 47 randomly selected Thai individuals was determined and the result of LD analysis is shown in Figure 1. The LD structure was generally similar to that of a population with European ancestry (HapMap CEU, data not shown). The rs245178 was found in a large LD block spanning 43 kb in our Thai population. To narrow the region containing the responsible SNPs, we selected the rs1421746, rs698171, and rs698172, which were in moderate LD with the rs245178, and tested for the association with schizophrenia. The rs698172 showed the strongest association (*P* = 0.003, under the dominant model) and the minor T allele was found to have lower frequency in patients than in controls (OR = 0.61) (Table 1). Sex and age adjusted- OR and 95% CI of carriers of the each minor allele were estimated as 0.50 and 0.30–0.84 for both SNPs. We also tested the rs248707, rs6959787, and rs7711139, which were located in the *CTXN3* gene and did not show strong LD with the SNPs tested above. The rs248707 and rs7711139, which possibly had functional consequences for the *CTXN3* gene, did not show a significant association with schizophrenia (*P* = 0.325 and *P* = 0.528, respectively). Marked deviation from Hardy-Weinberg equilibrium was observed for the rs6959787 (*P =* 0.004 in the patients; *P* = 0.0009 in the controls) and thus, association analysis was not performed for this SNP. Among the seven SNPs tested, the rs245178 and rs698172 remained significant at 5% level after the Bonferroni correction for multiple SNP testing (*P* < 0.0071). Degree of dominance *h* for rs245178 and rs698172 were −0.50 and −0.51, respectively. These values fell into 0 < |*h*| < 1, supporting that the minor allele of these two SNPs were dominant [14]. LD status of the 7 SNPs in the patients and in the controls are shown in Figure 2. Neither *D’* nor *r*^*2*^ values showed striking differences between the patients and controls.

### Haplotype analysis

We reconstructed haplotypes of rs245178 and rs698172 and tested the differences in haplotype frequencies between patients and controls (Table 2). A total of four haplotypes were observed in the studied population. Frequencies of these haplotypes were significantly different between the patients and the controls (*P* = 0.0447). Moreover, a haplotype consist T allele of rs698172 and G allele of rs245178 was significantly underrepresented in patients (in Table 2; Haplotype 2 versus Haplotype 3, *P* = 0.005, OR = 0.58).

**Table 2:** Haplotype analysis

	**rs698172**	**rs245178**	**Patient**	**Control**
Haplotype1	T	A	0.09(0.02)	0.10(0.02)
Haplotype2	T	G	0.29(0.03)	0.39(0.03)
Haplotyep3	C	A	0.58(0.03)	0.46(0.03)
Haplotype4	C	G	0.04(0.01)	0.04(0.01)
	P^*^	**OR (95%CI)**		
Overall	0.04			
Haplotype 2 versus Haplotype 3	0.005	0.58(0.43–0.79)		

*P values in *χ*^2^ test are shown.

For each haplotype, relative frequencies and the standard deviation (in parentheses) are shown.

## Discussion

In the present study, a total of four SNPs in *CTXN3-SLC12A2* region—rs245178, rs698172, rs1421746, and rs698171 were shown to be significantly associated with a risk of schizophrenia in a Thai population that is consistent with the previous GWAS report [8]. Another GWAS-discovered SNP, rs245201, showed a marginal association with schizophrenia (*P* = 0.052, under the dominant model). In a population of European ancestry (HapMap CEU panel), rs245201 was in absolute LD with rs698172, but this was not the case in our Thai population (*r*^*2*^ = 0.5). The differences in LD structure would explain the apparent contradiction between these studies. Furthermore, the rs698172, which was separated by 45.5 kb from the rs245178, showed the strongest association with risk of schizophrenia in the Thai group. The minor T allele of rs698172 was shown to reduce the risk of schizophrenia. The haplotype-based association analysis revealed that the frequency of haplotypes of the rs698172 T and rs245178 G alleles was significantly lower in patients (*P* = 0.005), and the odds ratio was smaller than those of rs698172 and rs245178. Further verification is required to determine whether this haplotype effect is the actual influences susceptibility to schizophrenia. The associations detected for the rs245178 and rs698172 were considered to simply reflect LD of these two SNPs. The rs245178 and rs698172 were located in the intergenic region between *CTXN3* and *SLC12A2* genes, and it is still unclear whether functional elements affecting gene functions exist in this region.

CTXN3 protein is a brain-specific integral membrane protein, highly enriched within the cortex [18]. It shows 43% homology with cortexin (CTXN 1), which is highly enriched in the cerebral cortex and is probably an integral membrane protein playing a role in the developing and adult cerebral cortex [18]. It is expected that CTXN3 is also a member of the integral membrane proteins and is relevant to the development and function of the cerebral cortex. Another candidate gene in this region, *SLC12A2*, was expressed in various human tissues, including brain [12]. *SLC12A2* was highly expressed in developing cortical neurons and promotes the accumulation of chloride ion (Cl^-^) in neurons [19]. Moreover, SLC12A2 protein facilitates seizure in the developing brain [19]. The significant associations between SNPs and risk of schizophrenia suggest potential roles of these two genes in the pathogenesis of schizophrenia.

## Conclusions

The present study showed that two SNPs in the *CTXN3-SLC12A2* region, rs245178, and rs698172 are associated with risk of schizophrenia in Thai population. These SNPs were located in intergenic region with unknown function. Further study, it would be of interest to identify genetic variation around these SNPs affecting the expression pattern of the *CTXN3* and *SLC12A2* genes and test the association with risk for schizophrenia.

## Competing interests

The authors declare that they have no competing interests.

## Authors’ contributions

BP and KN participated in the study design, performed the genetic studies, statistical analysis and prepared draft of the manuscript. SI gave critical comments on the manuscript. WT investigated and diagnosed the control and patient according to DSM-IV criteria. WS participated in study design, concluded the results, and revised manuscript. All authors read and approved the final manuscript.

## Supplementary Material

Additional file 1 Primers for high-resolution melting analysis. Click here for file
