# Supplementary material for: Association of CTXN3-SLC12A2 polymorphisms and schizophrenia in a Thai population
Source: Behav Brain Funct. 2012 May 29;8:27. doi: 10.1186/1744-9081-8-27 (PMC3433332; doi:10.1186/1744-9081-8-27)
Supplement: Additional file 1 — Primers for high-resolution melting analysis. [file 1744-9081-8-27-S1.doc]

**Supplementary materials**

Primers for high-resolution melting analysis

| SNPs | Primer sequences |
| --- | --- |
| rs245178 | F:CCACCTCTACAGTTGTTGGTAT |
|  | R:CGTCACTTCTTACAGATGTTTGC |
| rs245201 | F:CCTCCTTATACTACATGCCATC |
|  | R:ATCCTTCTACCTAAACAACCATTC |
| rs248707 | F:GTCCTGAGCAGAATGAGGAA |
|  | R:CAAGAAACTACTTTGGGTCCAT |
| rs698172 | F:CATCTCTTGAGGGAAAGTCG |
|  | R:ACACGCTAAGGCAACTAC |
| rs6595787 | F:AAATCATCCTGTTTCATCAC |
|  | R:AAGCCTAGAATACTATACCGA |
| rs7711139 | F:CATTAATATCCTCTAAATTCCTAAAGAGC |
|  | R:GGAAGTAAGATTGGGATAAAAATTTGAC |
| rs1421746 | F:TGCCTCCTTTGCAGAAAAT |
|  | R:GCTTTTTAGAGGGAATTTGCT |
| rs698171 | F:GCTGTTAGTCATCACTCAATGTTAC |
|  | R:ACACATATACACACACACTGGAA |
| rs1421746 | F:TGCCTCCTTTGCAGAAAAT |
|  | R:GCTTTTTAGAGGGAATTTGCT |
| rs711360 | F:GGTTTAAAGAAACAAATAGCATGG |
|  | R:ACTGTAGGCTGGAAAAATATTAGA |
| rs245314 | F:TGGAAATATGAAGTGTCCATA |
|  | R:GGGACTAAATATAATACCC |
| rs245311 | F:TTAAATTTATCTAAACACAGA |
|  | R:ATTTCCATAGGTTGCCAAAAT |
| rs245310 | F:CATATTTTGGCAACCTATGGA |
|  | R:AGGTGAGTTGCTGAAAACA |
| rs9285907 | F:GTTGATAATAGATAATCAGCTAA |
|  | R:ATGATAGAGTAGAGGTATAAAT |
| rs151849 | F:AGAGAAAAGCAGATTTTTAAG |
|  | R:GTTTTGGAACCATTTCATT |
| rs6874357 | F:CTTAGGTTAACTTCTCTGAGCTG |
|  | R:AGTGTGATGAAATACAGTATCCC |
| rs245199 | F:CTTTCTCTCAGGGGAAAGATT |
|  | R:TGAACATCAGTCATTCCCTT |
| rs245195 | F:AGGACTTGACCAAAAGCTA |
|  | R:AACCTGTGTACCTACTTCT |
| rs245192 | F:GTTGGGACAACCTGGGA |
|  | R:CCACAAACTTCTGTGGC |
| rs245191 | F:TTTGGAAGTACACAACACC |
|  | R:CCCATAGTCCTTCTTAGAGAT |
| rs1579284 | F:CAATCTCCATTTCTAATTCTAC |
|  | R:ACTCCAACAGAGGAAGTAA |
| rs181746 | F:AAGAGTTACCAAGATGTGACAA |
|  | R:ATGTCAAGCAAGTCTATCAGT |
| rs245186 | F:TACTGGGATAAGCAAGACAATCAT |
|  | R:AGTCTGTTTCCATAACTAGACTCT |
| rs6864687 | F:TAATGAGCTGTATCCTCTC |
|  | R:CATGTTTTTCTCACCGGGTTA |
| rs151879 | F:CCCAGCCATTATATATAGTTATT |
|  | R:CAACATCACACTCTGAAATTA |
| rs245179 | F:TAGAGGTGGGGCTAAGAAAC |
|  | R:GACAGAAAGTGCCCAAAGG |
| rs245181 | F:CAACAAACAACCTGACTCA |
|  | R:AAACTGAGACTTTCCTCCA |
| rs245183 | F:TAAATTCCTATTACCGTGC |
|  | R:AATACATGATTTCCTCCCT |
